# Comparative exploration of whole-body MR through locally rigid transforms

**DOI:** 10.1007/s11548-013-0820-z

**Published:** 2013-06-01

**Authors:** Oleh Dzyubachyk, Jorik Blaas, Charl P. Botha, Marius Staring, Monique Reijnierse, Johan L. Bloem, Rob J. van der Geest, Boudewijn P. F. Lelieveldt

**Affiliations:** 1 Department of Radiology, Leiden University Medical Center, Leiden, The Netherlands; 2 Intelligent Systems Department, Delft University of Technology, Delft, The Netherlands

**Keywords:** Whole-body imaging, MRI, Registration, Comparative visualization

## Abstract

*Purpose*   Whole-body MRI is seeing increasing use in the study and diagnosis of disease progression. In this, a central task is the visual assessment of the progressive changes that occur between two whole-body MRI datasets, taken at baseline and follow-up. Current radiological workflow for this consists in manual search of each organ of interest on both scans, usually on multiple data channels, for further visual comparison. Large size of datasets, significant posture differences, and changes in patient anatomy turn manual matching in an extremely labor-intensive task that requires from radiologists high concentration for long period of time. This strongly limits the productivity and increases risk of underdiagnosis.

*Materials and methods*   We present a novel approach to the comparative visual analysis of whole-body MRI follow-up data. Our method is based on interactive derivation of locally rigid transforms from a pre-computed whole-body deformable registration. Using this approach, baseline and follow-up slices can be interactively matched with a single mouse click in the anatomical region of interest. In addition to the synchronized side-by-side baseline and matched follow-up slices, we have integrated four techniques to further facilitate the visual comparison of the two datasets: the “deformation sphere”, the color fusion view, the magic lens, and a set of uncertainty iso-contours around the current region of interest.

*Results*   We have applied our method to the study of cancerous bone lesions over time in patients with Kahler’s disease. During these studies, the radiologist carefully visually examines a large number of anatomical sites for changes. Our interactive locally rigid matching approach was found helpful in localization of cancerous lesions and visual assessment of changes between different scans. Furthermore, each of the features integrated in our software was separately evaluated by the experts.

*Conclusion*   We demonstrated how our method significantly facilitates examination of whole-body MR datasets in follow-up studies by enabling the rapid interactive matching of regions of interest and by the explicit visualization of change.

## Introduction

The rapid progress in MR scanning technology increasingly enables imaging the whole body, and clinical applications of whole-body MRI are rapidly emerging [1–3]. For instance, vascular imaging protocols have been developed that enable a vascular checkup and risk assessment for cardiovascular disease [4, 5]. These do enable imaging not only the entire vascular system (including carotids, aorta, renal arteries, and leg vasculature) [6], but also the amount of body fat and its distribution over the body [7, 8]. The presence of excessive fat in the abdomen is an important risk factor for the onset of vascular diseases, and in combination with the vasculature diagnostic data, whole-body MR may provide a good assessment of the status and risk factors for a patient. Also for oncological applications, diffusion-weighted and Short TI Inversion Recovery (STIR) whole-body MR protocols have been developed for the detection of cancerous lesions [3, 9–11] and for cancer staging based on, for instance, bone marrow involvement in hematological cancers. These protocols have been shown to enable evaluation of treatment effect, shortly after the onset of chemotherapy regimen or radiotherapy.

The introduction of these new whole-body MR imaging techniques provides important clinical benefits. However, there are several technical challenges inherent to whole-body imaging that so far have only been sparsely addressed due to the relative novelty of whole-body MR acquisition. In particular, comparative visual analysis of whole-body MR follow-up data is difficult. Although the number of methods available for medical image visualization is constantly increasing and even whole-body explorative visualization is becoming a reality [12], only very few groups have addressed the problems inherent to comparative whole-body scanning: the inevitable differences in patient posture between follow-up scans, in combination with the massive amount of data. This, in particular, is a problem in radiological evaluation of metastatic cancers, where metastases can appear throughout the body. For instance, breast cancer, prostate cancer, and Kahler’s disease tend to metastasize to bone tissue, where lesions can occur in each individual bone. Such patients may undergo periodic evaluation using whole-body MRI. In reading, comparing and reporting these scans, the radiologist has to answer three main questions: 1) Are there any new lesions compared to the previous scan in any of the bones of the whole skeleton?, 2) How did previous lesions evolve over time as a result of disease progression or treatment?, and 3) Is there risk of bone fracture due to progressive bone loss?

To answer these questions, the radiologist has to perform a side-by-side comparison of each individual bone from head to toe, for two (or more) follow-up scans. This is a cumbersome process, because the posture of the patient usually is different between the scans. This greatly complicates localizing corresponding locations with respect to bone anatomy. In addition to comparing both $\text{ T }_1$-weighted (T$_1$W) MR images for anatomical detail, other MR sequences, e.g., STIR images that provide better definition of the cancerous lesions [9], often have to be investigated. As a result, the reporting process of a follow-up whole-body scan may easily take the radiologist 1 h, since it is particularly important to make sure that no lesions are missed due to the detrimental consequences for patient prognosis and quality of life. As such, there is a great demand for more automated visual analysis methods that aid in identifying the identical anatomical location and slice view in follow-up whole-body MR scans.

**Fig. 1:**
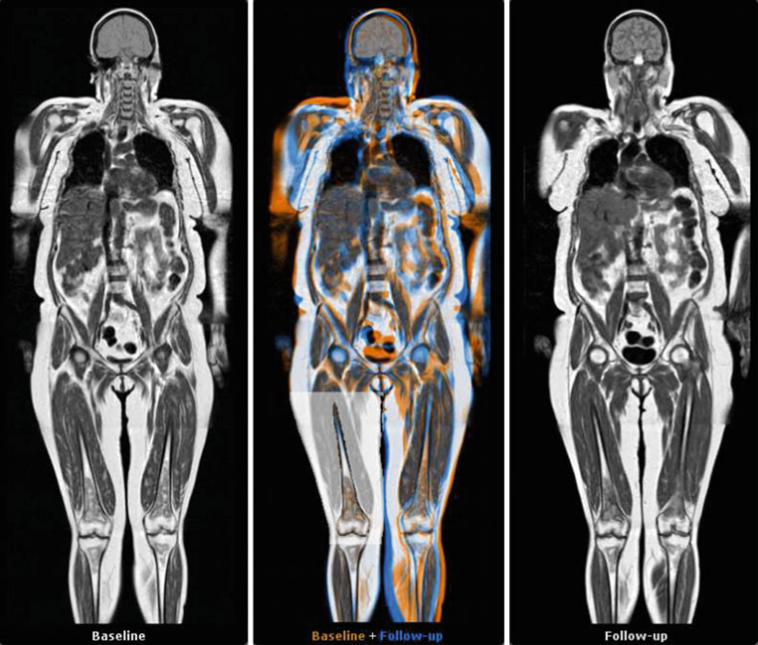
A slice view showing a follow-up whole-body MRI study of a single patient. The baseline scan is shown on the *left*, the follow-up on the *right*. The *center* view provides an *orange–blue* fusion of baseline and follow-up. The *right* upper leg, indicated by the surrounding area converted to grayscale for visualization purpose, is the region of interest. A single click there has segmented part of the bone, resulting in a rigid transform that maps the follow-up to the baseline in this region. Note that even though the rest of the body is aligned differently, the local correspondence is well maintained, allowing for a coordinated comparison within a region of interest
- Besides visualizing the synchronized side-by-side baseline and matched follow-up slices, we present four ways to facilitate a details-on-demand-based visual comparison of the two datasets: 1) The deformation sphere [13] is a spherical cloud of points that follows the mouse cursor, with each of the points being warped by the local deformation field. The sphere assists comprehension of the actual local deformation field. 2) The color fusion view combines the baseline and follow-up in one view by making use of two (perceptually motivated) color channels. 3) We render a set of uncertainty iso-contours around the current region of interest [14], explicitly visualizing the approximation error of the locally rigid transform. 4) We have integrated a magic lens [15] that displays an alternate MR modality so that areas of change can be further studied.
- Finally, we investigate the role and utility of our methods toward improving the radiologists’ workflow in the assessment of tumor burden in patients suffering from Kahler’s disease by means of a case study with two experienced skeletal radiologists.

In this work, we present several methods to facilitate the visual analysis of whole-body MR data for evaluation of oncological follow-up studies. Our contributions are threefold:

- We present a novel comparative visualization approach based on interactive derivation of locally rigid transforms in a region of interest from a pre-computed whole-body deformable registration. These are subsequently applied to the coordinated visualization of baseline, follow-up, and fused whole-body slices. As bones are inherently rigid, these transforms form a good basis for compensating for posture differences by matching follow-up to baseline by simply selecting one bone at a time. Using this locally rigid transform approach, baseline and follow-up anatomical regions can be interactively matched, based on a single click of the mouse in the anatomical region of interest; see Fig. 1 for an illustration.

In this paper, we focus on the analysis of oncological follow-up whole-body MR data. However, the presented methods can be used for other applications as well, given a dense non-rigid registration between two follow-up time points.

## Related work

The work presented in this paper focuses on enabling the efficient comparative visualization of two whole-body MRI datasets, being mostly baseline and follow-up datasets of the same patient. Our technique involves image registration, interactive segmentation, and comparative visualization. In this section, we briefly survey related work on image registration, whereas we go into more depth summarizing comparative visualization, as that is where the contributions of this work lie.

Image registration refers to the process of transforming different images into one coordinate system, often for the purpose of comparison. More specifically, image registration refers to the derivation of a transformation that maps a moving image onto a fixed image, so that pixels from the two images can be directly compared. A number of excellent surveys have been written on the topic [16–18]. In this work, we make use of an efficient implementation of non-rigid registration with mutual information [19] and B-splines [20, 21]. This specific registration approach is used often in deformation-based morphometry [22], i.e., the study of biological shape changes in image data.

Comparative visualization refers to the visual representation of multiple data sources with the express purpose of studying the similarities and differences in those data sources [23]. Examples include side-by-side comparison [23, 24], coupled side-by-side views [25, 26], image superposition [27], and more refined methods such as that by Schneider et al. [28], where they used contour trees for matching and comparing different scalar fields in a flow dataset. Recently, Rieder et al. [29] have developed a tumor visualization tool that incorporates lesion segmentation and manual local registration of two regions of interest. The work by Kok et al. [30] is related to this, in that the skeletons were extracted from small animal imaging datasets and registered, taking into account joint articulation, allowing for the direct comparative visualization of the sub-volumes containing the various limbs.

Image superposition, for example, checkerboard or color fusion displays, is quite popular in medical practice and research [27]. The work in this paper combines a color fusion display with a new type of interactive model-based comparison, where the user implicitly specifies the model by clicking on a part of the anatomy that interests him/her. Based on this expression of interest, the method calculates a transformation that is optimized for the comparison of the region around the selected point. The method by Kok et al. [30] offered a top-down approach to comparison, in which exploration of each bone of interest requires fitting the entire skeleton atlas to the data. Our method, on the contrary, allows exploration of the complete body volume from its local parts. It thereby utilizes interactively specified subsets of the skeleton, which is more suitable to the radiological workflow than atlas-based methods.

The contribution of this work is the interactive matching of datasets according to user-specified regions, and especially the real-time derivation of this matching based on the real-time segmentation and locally rigid approximation of a global deformable registration. Furthermore, this is the first application of comparative visualization to whole-body MRI datasets.

## Integrated exploration through locally rigid transforms

### Locally rigid registration

In the design of our visual analysis, we aim to maximally leverage the conventional workflow of the radiologist in reporting follow-up whole-body MR. This requires visual side-by-side comparison of two volumes, the baseline and the follow-up. For the coordinated display of the two volumes, it is necessary to establish correspondence between all voxels of the first dataset and those of the second one, which is achieved by image registration. Due to inevitable posture differences between the two scans and other possible differences, like weight gain or loss by the patient during the time period between the scans, rigid alignment of the complete volumes is impossible. Non-rigid registration, on the other hand, is able to align the volumes with reasonable quality. However, this often happens at the cost of producing unnatural deformations of the rigid parts of the body, since the global aim of deformable registration is to compensate for differences between scans, including both posture and anatomical changes. Whereas our main goal is to enable the visual detection of cancerous lesions, while getting rid of posture differences. Hence, for performing accurate comparison of two regions of interest, they have to be aligned in a *locally rigid* fashion. This is especially so in the case of Kahler’s disease, where the radiologists are primarily interested in inspecting the bones where the disease metastasizes. By restricting ourselves to rigid transformations, we get rid of rigid bone posture differences while explicitly retaining differences caused by changing lesions. Last but not least, such rigid matching of the regions of interest is much better accepted by radiologists, who find it more natural, hence trustworthy, than the deformable one.

Thus, alignment of the baseline and the follow-up whole-body volumes reduces to finding a way to establish rigid correspondences between two local regions of interest, subjected to the constraint that these correspondences should be defined for every possible region of interest. This constraint is motivated by the observation that Kahler’s disease can metastasize on every bone in the human body, from head to toe. In practice, resolving even such a simplified problem is difficult. Interactive alignment of the two regions is infeasible since it requires their segmentation in both the baseline and the follow-up.

To overcome the mentioned difficulties, we have developed an interactive approach that estimates the local rigid transformation from the pre-computed global deformation. We utilize this transform to fully automatically align both datasets in the area of interest, in real time. Our approach has several important properties that make it suitable for side-by-side comparison as performed in the conventional radiological workflow:

- It is based on *rigid image matching*: no distortions are introduced.
- It requires *minimal user interaction*: one mouse click is sufficient for aligning the regions of interest.
- It *does not require manual segmentation* of the regions: the latter is calculated automatically, starting from the user-provided seed point (one mouse click). Subsequently, the corresponding region of the follow-up scan is obtained from the pre-computed global deformation.
- It is *fast*: the more time-consuming operations are performed at the pre-processing stage. Segmentation of the structure of interest, estimation of the rigid transform in the area, and re-alignment of the follow-up volume are performed on-the-fly. Derivation of the locally rigid transform from the global deformation field is done with a closed-form relation, which will always be significantly faster than any iterative on-the-fly locally rigid registration technique.

The following sections describe the efficient computation of the localized transforms and our user interface prototype that incorporates these into a radiological workflow.

### Computing localized transforms

**Fig. 2:**
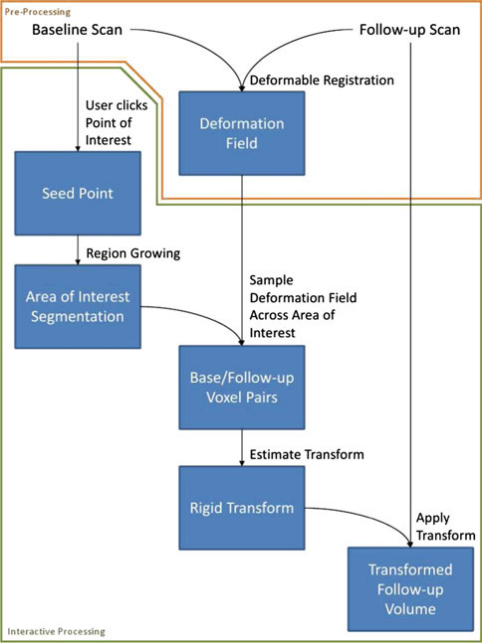
The processing pipeline for deducing a locally rigid transform, given a point of interest. The *links* in this diagram represent the operations, while the *boxes* represent the resulting data. The pre-processing operations are marked in *orange*

Figure 2 shows the steps required to compute this locally rigid approximation of the deformation field surrounding a given point of interest. First of all, the point of interest is used as a seed point for a region growing algorithm, creating a coarse segmentation of the object of interest. Subsequently, the voxels belonging to the segmented object are matched to the corresponding voxels in the follow-up image, by using the pre-computed deformable registration. Once the locations of all segmented voxels are known both in baseline and follow-up coordinates, these point-pairs can be used as landmarks to estimate the rigid part of their transformation. Finally, the resulting rigid transform is applied to the follow-up volume in order to align it with the baseline volume. The following sections explain the steps of the algorithm in more detail.

#### Area of interest segmentation

To generate the vectors that are used to approximate the rigid transform, we need a set of points that are likely part of the structure of interest. We do not necessarily need to accurately segment the entire structure, as long as a large majority of the points are correctly segmented. Points that are falsely identified as belonging to the structure (false positives) are more of a problem than points that are falsely identified as belonging to the background (false negatives). The former will affect the rigid transform estimation more severely as they belong to the image part where the rigid approximation is less valid.

Region growing is widely used for the segmentation of medical images, in particular, skeletal structures in MR data [31, 32]. Here, we have adopted a confidence connected region growing method [33]. The advantage of this implementation is that it is fast and has few operational parameters, with which it can be set to return more or fewer points based on a confidence interval. The latter makes it an ideal candidate for our task, as we can influence the ratio between false positives and false negatives.

**Fig. 3:**
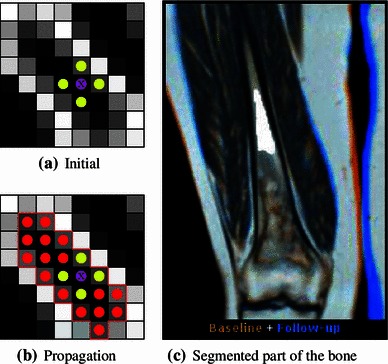
Region of interest segmentation by confidence connected region growing. The initial condition around the seed point and the region growing are shown on the *left*, while the resulting segmentation of a part of a bone is seen on the *right* (*white mask*)

The segmentation process is illustrated in Fig. 3. The illustration shows the two-dimensional case, but we employ a three-dimensional version of the algorithm. Surrounding the point of interest (indicated by the purple dot), a small spherical region is created (the yellow dots), which is assumed to be within the object of interest. Based on the mean and variance of the points within this region, the initial selection is expanded incrementally with neighboring voxels that fall into the same distribution with high probability [33]. For execution of the algorithm, we have used the following parameter values: *multiplier* $=$ 1, *number of iterations* $=$ 1, *initial neighborhood radius* $=$ 2, and *replace value* $=$ the maximum intensity value in the data.

To constrain the computation to an anatomically reasonable area of interest, we constrain the region growing to an empirically selected $10~\mathrm{cm} \times 10~\mathrm{cm} \times 10~\mathrm{cm}$ box around the point of interest. This prevents the algorithm from taking up valuable processor time in the rare cases where it accidentally over-segments and floods into neighboring regions.

#### Baseline and follow-up point matching

To perform the rigid transform estimation step, all voxels that are inside the selection need to be mapped to voxels in the follow-up image. The dense deformation field generated by the deformable registration step during pre-processing provides such a mapping. By sampling the deformation field for all voxels included in the area of interest, we extract pairs of baseline and corresponding follow-up coordinates. As the transformation of the anatomical object of interest is captured in this set of point-pairs, they form the basis for the transform estimation in the following step.

#### Rigid estimation by landmark transform

The landmark transform is often used to calculate the rotation component of absolute orientation [34]. The method uses unit quaternions to find a non-iterative closed-form solution, which makes it accurate and highly efficient.

**Fig. 4:**
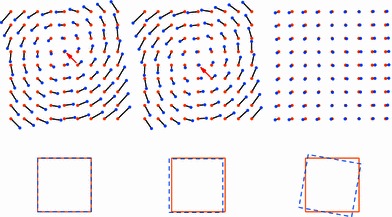
Deformation field of baseline (*orange*) and corresponding follow-up (*blue*) positions, during the rigid transform extraction process. From *left* to *right*: original field, field after removing translation, field after removing both translation and rotation. The *bottom row* shows the transforms that have been applied to the point sets as rectangles in the corresponding colors

As shown in Fig. 4, the set of point-pairs can be decomposed into a rigid transform part and a non-rigid part. Starting with the base situation, on the left, where no transform has been applied to either dataset, we note from the length of the black lines that the discrepancy between the two fields seems rather large and fairly structured. After applying the resulting transformation, the discrepancy between baseline and follow-up points is now reduced to only the non-rigid components, shifting the visual focus to the actual deformations.

Note also how between the left and middle figure, the apparent center of the rotation, indicated by red arrows in Fig. 4, is displaced, even though only a small translation has been applied. This visual deception illustrates that the removal of the rigid transform component is a key element in the visual assessment of deformations.

#### Application of found rigid transform

Once the transform has been found, it is applied to the follow-up volume. The volumes are only sampled when slices have to be generated for viewing, keeping into account their relative orientations.

### User interface

To test how our methods fit within a radiological workflow, we have made a prototype implementation that encompasses the entire visual examination workflow. The user interface of the application is split into three main parts. To closely mimic the normal radiological workflow, the main components are three side-by-side slice views.

For consistency, the views are structured in a fixed way so that the baseline image is always on the left, and the follow-up is always on the far right. The middle view shows a fusion of both images, to further ease the comparison (see “Color fusion view”).

Each view shows a two-dimensional slice through the three-dimensional volume. By right-dragging, the user can move in the image plane, while center-dragging is used to navigate in the slice direction. The mouse wheel is used for zooming, as to enlarge details. Moving in one view will always keep the other views synchronized to show the exact same spatial area. Navigation of these slice views is always linked between all three views.

Once a point of interest is found in one of the images, a mouse click suffices to align the follow-up image to the baseline, using the locally rigid transform estimation surrounding the clicked point.

The slice views can optionally show a number of overlay components. These overlays will be explained in more detail in the following sections. To make the user interface as lean as possible during normal diagnostic procedures, the overlay elements can be toggled on and off by a set of checkboxes at the bottom of the screen, or by pressing the associated number keys.

#### STIR viewing

**Fig. 5:**
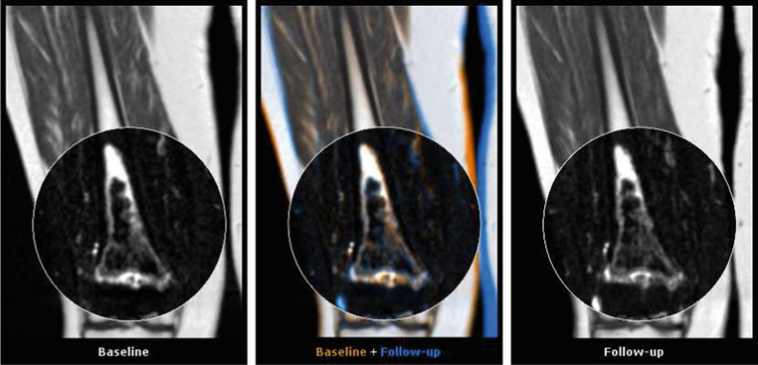
Slice view of the data, with the STIR lens overlay enabled. From *left* to *right*: baseline, color fusion of baseline and follow-up, and follow-up. The white circular lens, which follows the cursor, shows a secondary MRI modality, providing better definition of the cancerous lesions. The image shown inside is co-located with the surrounding data, which as such functions as an anatomical reference. The STIR lens also participates in the color fusion, so that changes in intensity in the secondary data are easily visualized

The data we have are inherently multi-modal, consisting of both T$_1$W and STIR volumes. The T$_1$W volumes are the default modality, as they provide the anatomical context. The STIR data are used for detailed examinations. To avoid cluttering the interface with many separate slice views, the STIR images are shown only in a details-on-demand fashion (see Fig. 5). To optimally profit from the co-localization of the two scans, we have chosen to use a magic lens to display the STIR images as an overlay [15]. The circular lens that follows the mouse cursor can be quickly toggled on and off. The position of the lens is synchronized through all three views, easing comparison of STIR values between baseline and follow-up.

#### Color fusion view

As previously mentioned, the radiologists are trained to work with grayscale slice views and construct a mental image of the anatomy of the patient from that. However, small changes in size and intensity are difficult to pick up using only a side-by-side view. This is why the central slice view shows a color-fused image of both follow-up and baseline. The colors we have chosen are orange and light blue, for two reasons. First of all, they are exactly complementary, so areas where the two intensities are similar become gray, leaving the areas where differences are present colored. Secondly, the colors can be perceived even by viewers with color vision deficiency.

**Fig. 6:**
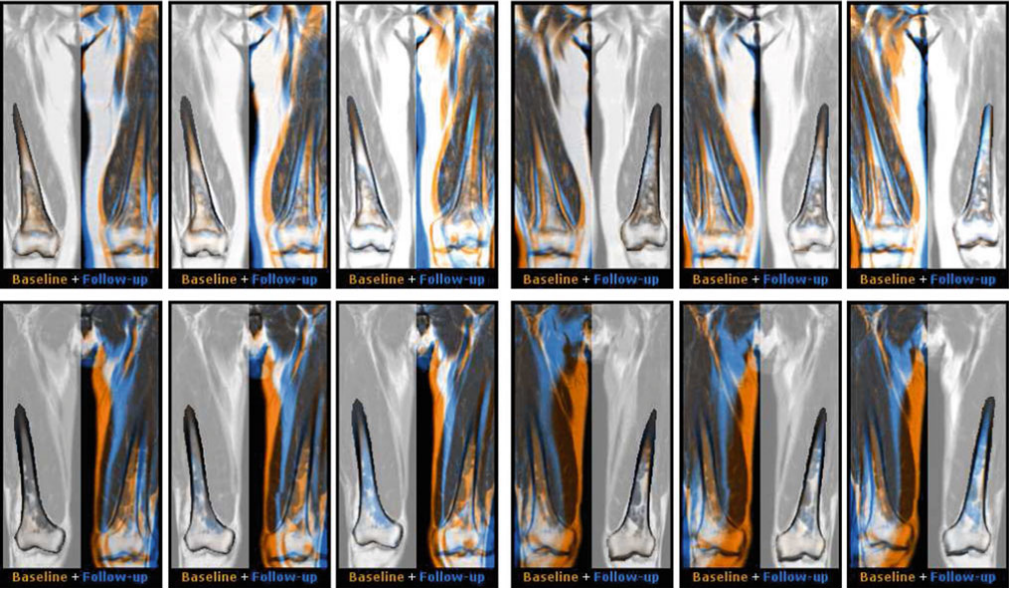
Color fusion of the baseline scan and three consecutive follow-up scans of two different patients (shown in different rows). For each patient, the data are displayed with either locally rigid alignment of the right femur (*first three columns*), or the left femur (*last three columns*); the area surrounding the region of interest is converted to grayscale for visualization purpose. Such color fusion view greatly simplifies visual assessment of changes between the baseline and the follow-up scans. For the first patient (*top row*), the *orange color* is dominating in the area around the lesions, indicating progression of the disease. For the second patient (*bottom row*), predominantly *blue color* is observed in the lesions area, with strongly increasing intensity of the *blue* channel between the first and the last baseline/follow-up pair of images. Such behavior indicates successful recovery of the patient

If intensities decrease locally, the color becomes orange, while an increase yields a blue color; see Fig. 6. Small misalignments are easily spotted by the blue border on one side and the orange border on the other.

#### Deformation sphere

**Fig. 7:**
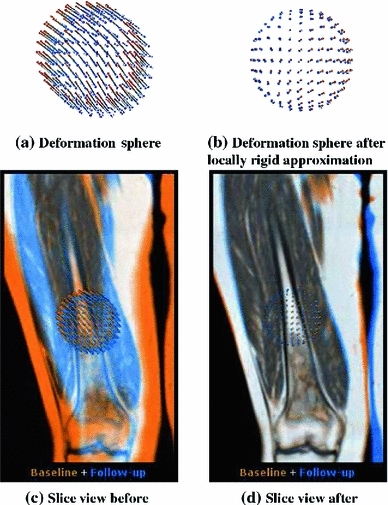
A set of deformation vectors for a sphere. The *orange* points correspond to the baseline (a sphere) while the *blue* points correspond to the sphere warped deformably to the follow-up coordinates. The longer the lines, the more distortion has occurred. On the *left*, a purely deformable registration, while on the *right*, a locally rigid transform has been estimated, which has been subsequently applied. As a result, the deformation vectors on the *right* only show the deformations that are not explained by the rigid transform. The *top row* shows only the sphere, while the *bottom row* shows it in the context of the application

To inspect the 3D deformation field and also assess how well the locally rigid approximation matches the deformable registration, we introduce a visual feedback component in the shape of a 3D sphere, as shown in Fig. 7. The sphere is shown both in the baseline as well as in the follow-up coordinate system. The sphere is a unit sphere in the baseline coordinate system, but due to the non-rigid registration, its shape in the follow-up coordinate system is deformed (by the non-rigid deformation field). By moving the sphere around, subtle changes in the deformation field can be picked up by monitoring the shape of the deformed sphere.

In the central fused view, a combined visualization is used to emphasize the difference vectors between the two spheres. Color-coded lines are drawn between each pair of points. The longer the lines, the larger the difference between the baseline and follow-up coordinates, and the less accurate is the locally rigid approximation.

#### Uncertainty contours overlay

Since the rigid transform is generated with the selected object in mind, the quality of the approximation will slowly diminish as the distance to the object gets larger. For rigid structures, such as bones, the approximation will be accurate across greater distances than for deformable structures, such as the internal organs. To indicate the accuracy of the rigid estimation, we propose a radially generated boundary around the point of interest, which indicates the absolute difference between the deformable transform and its rigid approximation.

**Fig. 8:**
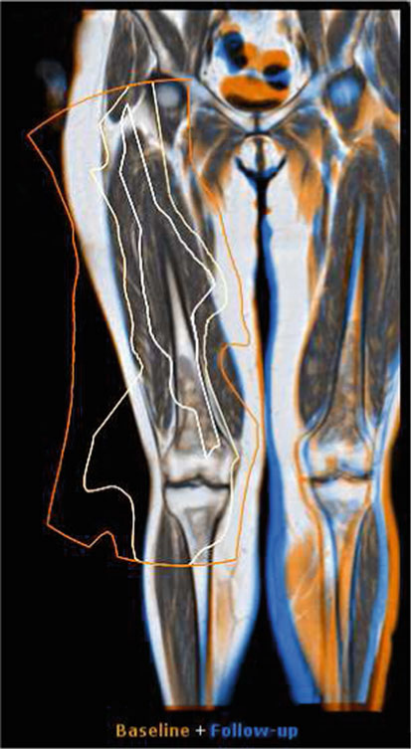
Uncertainty contours showing the accuracy of the rigid approximation, a section of the upper left leg. Each contour delineates a fixed error margin, from inner to outer, respectively, of 3, 6, and 9 mm

We introduce three lines, functioning as nested contours, that each correspond to a pre-set allowable error, as seen in Fig. 8. In the examples shown, the errors are 3, 6 and 9 mm from inner to outer. These contours are kept as a radial structure, so that it is immediately clear what the uncertainty around the point of interest is. In our software, these contours are centered around the mouse position, allowing interactive exploration of the current transform, but they can also be statically linked to the pre-selected point of interest. Note that the error contours stretch further along the bony structures, indicating the matching is accurate as is to be expected along such a rigid structure.

## Implementation and performance

Whole-body MR datasets can be quite large and therefore processing these can become a computational burden when multiple scans have to be compared. A whole-body scan with an isotropic voxel size of 1 mm can easily reach a size of $512 \times 350 \times 1{,}800$ voxels, resulting in a 16-bit data volume of over 600 megabytes.

The prototype application is implemented in C++, using the Insight Toolkit for segmentation and volume handling [33]. All drawing routines, such as slice rendering and overlays, use the OpenGL API, to ensure portability across platforms.

The patients^1^ for our study are scanned coronally at a $1~\mathrm{mm} \times 1~\mathrm{mm}$ in-plane resolution, with 5 mm between slices to reduce the total scan-time; for more details on scanning parameters, we refer reader to [35]. During pre-processing, the volumes are resampled to an isotropic resolution of $1.5~\mathrm{mm} \times 1.5~\mathrm{mm} \times 1.5~\mathrm{mm}$ due to memory limitations on the PC on which the prototype was implemented and tested.

### Pre-processing

The pre-processing routine consists of two major steps. Firstly, the data of each scan are intensity inhomogeneity corrected and stitched into a complete volume. Secondly, the generated volumes are registered pairwise, each follow-up onto its corresponding baseline.

#### Stitching and inhomogeneity correction

Whole-body MRI scans are usually made in multiple acquisition stages [1]. Since the field of view that can be imaged by the scanner is limited, the patient has to be repositioned. Much like panoramic photography, several slightly overlapping acquisitions are joined together into one large volume to extend the field of view. The patient is placed in the scanner on a movable table, which can move automatically after each acquisition. In the data used in this work, the patients were typically scanned in six stages, with a 5 cm overlap between succeeding volumes.

Using the spatial coordinates that are embedded by the MRI scanner, the volume of each acquisition stage is positioned into the patient-space. Once all transforms are known, a resampling operation is performed to create the whole-body volume.

Due to field inhomogeneity, the signal intensity can vary gradually within an acquisition. These intensity changes are mostly visible at the boundaries of the field of view of the scan. We construct a smooth approximation of the bias field and subsequently use it to suppress these intensity variations. For calculating the bias field, we have developed an inhomogeneity correction algorithm [35] based on the fuzzy-c-means clustering [36]. The bias field correction in our algorithm is performed jointly on the T$_1$W and STIR image stacks, and the intensity in the overlap areas is used to constrain the optimization. Such bias correction is rather conservative as it does not suppress intensity changes that are induced by the lesions. In addition to the estimated bias field, this algorithm also returns the probability maps for each of the main tissue classes. Finally, intensity of all scans is standardized by mapping it onto the same range of grayscale values.

#### Registration

The deformable registration is implemented using elastix [37]. Using this software library, we have constructed a multi-level registration approach. Initially, the coarse alignment is estimated using an affine transform. After this initial registration, an elastic registration takes place. This elastic registration makes use of a multi-resolution process (3 resolutions), in which a deformable B-spline grid is incrementally refined. In the last pass, the finest level, the B-spline grid has a spacing of 12 mm. As a similarity metric to be optimized by the registration, we have chosen to use a Mattes mutual information [19] (32 histogram bins), although other metrics may also be suitable for this purpose. A stochastic gradient descent optimizer [38] was selected, using 1,000 iterations.

The anatomical T$_1$W scans are used for the registration, as they provide the most amount of detail. Additional MRI sequences, such as the STIR volumes, are typically acquired interleaved with T$_1$W and are therefore already in alignment with the anatomical data.

After performing the registration, the average mutual information value on 32 whole-body volume pairs has increased from $0.32\pm 0.12$ to $0.90\pm 0.08$, with average increase of $0.56\pm 0.14$ on a pair of registered volumes.

### Timing and performance

We have profiled the prototype application on an HP Z400 workstation, with a quad core Intel Xeon W3530 processor at 2.8 GHz and $6$ gigabytes of RAM, and an Nvidia Quadro FX1800 videocard.

The intensity inhomogeneity correction and stitching together take from 10 to 15 min depending on the total image size, and registration takes less than 5 min, which is fast enough for our purpose, especially considering the MRI scan takes almost an hour to acquire. The registration is sped up considerably by selecting a stochastic gradient descent optimizer [38], thereby only having to evaluate a small number of points during the incremental optimization.

At startup, the application needs around half a minute to load the data files for the selected patient to memory. This includes two T$_1$W volumes, two STIR volumes, and the deformation field.

All operations that are part of the interactive workflow (indicated by the green border in Fig. 2) are executed in less than 0.2  s. Updates during scrolling and mouse motion, such as the deformation sphere updates, take less than 10 ms and therefore run at the full framerate of the display device. Refer to Table 1 for more detailed timings. At all times, an interactive framerate is maintained.

**Table 1:** Performance figures for preprocessing and the various stages of the pipeline (average of 48 datasets)

Operation	Time
Preprocessing (bias correction and stitching)	10–15 min
Preprocessing (registration)	$<$5 min
Sphere deformation	10 ms
Segmentation	100–200 ms
Slicing	100–200 ms

During use, memory usage peaks at around $2.6$ gigabytes. The memory usage of the application is largely dominated by the size of the deformation field, which is stored in floating point vector format, resulting in a storage cost of 12 bytes per voxel. If necessary, this could be further reduced by downsampling the deformation field, as it is usually quite smooth.

## Evaluation

To further validate the suitability of our prototype for the intended task, we performed a case study evaluation according to the guidelines set out by Yin [39]. We wanted to investigate how well our intended application, being the study of pathological changes in whole-body MRI, would be received by its main intended user group: radiologists.

### Case study

We formulated our main study question as: “How does the interactive whole-body MRI comparative visualization tool facilitate the study of bone tumor treatment or progression?” Our secondary question is similar but more generally framed: “How does the interactive whole-body MRI comparative visualization tool facilitate visual comparison in morphometrics?” As our case, we selected the application of our tool to four sets of whole-body MRI volumes by two radiologists experienced (together more than 40 years of experience as radiologist) with oncological whole-body MRI in the clinical workflow. Each set of the whole-body MRI volumes corresponded to the same patient and consisted of a baseline scan and multiple follow-up scans. In two different sessions, the radiologists each examined two different pairs of whole-body MRI. The radiologists were not involved in the development of the method, and their first contact with the prototype was during the case study, so their role as case study subject was justified.

To answer the case study questions, we decomposed them into a number of propositions that relate directly to the different components of our approach. During the case study, we investigated each of the propositions in turn. In the text, we refer to the users interchangeably as “radiologist”, “user”, and “domain expert”.

*Interactive locally rigid transforms enable the rapid matching of slices from baseline and follow-up*.

In the current clinical workflow, the radiologist scrolls through the baseline dataset, finds something interesting, for example, a tumor, then scrolls through the follow-up dataset trying to find the matching slice. This is mostly not possible, as the main slice directions usually do not align due to pose differences. The process is repeated a number of times, and the radiologist tries to construct a mental model of the changes, for example, growth or shrinkage of the tumor. Our approach for the matching of baseline and follow-up was deemed accurate and fast.

*The deformation sphere helps to clearly distinguish between areas of rigid and non-rigid change.*

One of the radiologists stated that the deformation sphere certainly helped to understand the deformations between baseline and follow-up, but that due to the usual time pressure and the fact that our approach offered other comparative visualization possibilities that she preferred, she would probably not make use of the sphere in practice. The other radiologist found the sphere not very intuitive, and thus, he considered it as having little added value for clinical use.

*The deformation sphere helps to gain insight into the nature of localized non-rigid changes, more so than standard side-by-side comparison for example.*

Both radiologists stated that they preferred the side-by-side and color fusion view over the sphere. They found that the color fusion view represented the match in an intuitive way and showed all differences in a single view, whereas the sphere required more interaction from the user. After more investigation, one of the radiologists stated that the deformation sphere could, indeed, be useful to study for example compression in the spine.

*The confidence boundaries clearly show where the rigid approximation is justified, and hence where the visual differences can be trusted.*

Although the users found the confidence boundaries to make sense, the color fusion display, in combination with their anatomical knowledge of the rigidity of the body parts, already supplied sufficient information concerning mismatches.

*The presented system speeds up whole-body MRI comparison both through the rapid matching of slices and through the subsequent explicit visual representation of local changes (deformation sphere/color fusion).*

Both domain experts agreed strongly with this proposition, stating that our method significantly sped up the localization of lesions, as well as the determination of their type.

*The STIR magic lens aids lesion assessment, in that lesion details can be better visualized with STIR, whilst (detailed) context is shown by the* $T_1W$.

The radiologists found this functionality very useful. Compared to the original workflow, where corresponding slices have to be sought across four different volumes (T$_1$W baseline and follow-up, as well as STIR baseline and follow-up), the ability to get a co-registered view of the STIR image was considered a major time saver. Visual inspection to find tumors is done by looking for lowered image intensity in T$_1$W and heightened image intensity in STIR, which can now be easily verified by switching on and off the overlay lens. Using this method, the users were easily able to find a number of bone lesions, that are otherwise quite hard to pick up, in one of the patients. Also, the possibility to use the T$_1$W magic lens on the STIR image was found useful.

*The color fusion view facilitates visual comparison between baseline and follow-up more than side-by-side views, as changes are visually emphasized.*

The color fusion view was much appreciated by the radiologists. In two cases, a diffuse infiltration of the bone marrow was visible as a blue region in the color fusion view and could be confirmed by the use of the STIR magic lens or by switching to the STIR view. One of the experts commented that she found the color fusion view, the deformation sphere, and the confidence contours to enable a three-part quality check of the data. Based on these, the distinction between differences due to pathology and those due to registration could be made.

### Summary

Both radiologists were clearly positive about the interactive whole-body MRI comparative visualization tool, agreeing strongly with our proposition that it sped up the localization and focused study of bone tumors. In this, the interactive matching was deemed to be centrally important, and the side-by-side and color fusion views to be most intuitive in studying the changes between baseline and follow-up.

The deformation sphere was seen by one user as useful in understanding the deformation and perhaps in studying, for example, compression in the spine. However, both users agreed that it would probably not find use in the clinical workflow due to the extra interaction it required, and mainly due to the fact that the other views sufficed for facilitating comparison. This conclusion can possibly be explained by the novelty of these concepts to both our users.

Based on this case study, we conclude that the tool facilitates the study of bone tumor progression, and visual comparison in morphometrics, primarily through the rapid interactive matching and the color fusion view.

## Conclusions and future work

We have presented a novel method for comparative visual analysis of whole-body MR datasets, focusing, in particular, on the local comparison of rigid structures. The prototype implementation is geared toward radiologists and uses accepted concepts from their standard workflow, while complementing it with the ability to perform coordinated visual assessment of disease progression.

The main contribution of this work is the development of the method that allows interactive alignment of follow-up and baseline images in a locally rigid manner based on the pre-computed global deformation. The mentioned properties of our method, being fast and rigid, are critical for successful assessment of tumor healing or progression.

We have also shown how our method is applied in a radiological case study targeting the progression of bone lesions in patients with Kahler’s disease. Although the evaluation of the developed methodology was performed on a single type of data, the developed methodology is very general and thus can be applied for various comparative studies. Its usage, in general, is not restricted to rigid structures: we expect that this approach is also fully or partially applicable to visualization of changes in, e.g., soft tissue. This, however, might require deriving local transforms with more degrees of freedom than the currently used rigid transforms to better reflect the physical properties of that particular tissue. In the future, we are planning to set up an additional case study that would allow quantification of several measures associated with our method, in particular, the inspection time gain per dataset provided by our method and the lesions detection rate. In this study, the results obtained with our tool will be compared with the ground truth in the form of clinical reports available for each of the scans.

A limitation of our application is that it currently only handles a single pair of datasets, while the underlying methods can easily scale to multiple timepoints. If a registration of each timepoint to the baseline scan is made, extending the number of parallel views should be a straightforward and welcome addition. This would allow for the examination of an anatomical structure of interest through an arbitrary number of timepoints.

To further reduce the memory requirements, as would be necessary when loading multiple timepoints, we propose to reduce the memory taken up by the deformation field. Instead of fully storing the deformation field, we could store only the B-spline control grid, vastly reducing memory usage. However, this would exclude the use of other nonparametric registration methods such as optic flow techniques.

The promising results in localizing and assessing changes in bone resorption encourage us to further evaluate the applicability of our method in future clinical studies. For example, the progression of tumor growth in the brain is difficult to assess visually, and non-rigid registration introduces unwanted deformations. Locally rigid transforms may be able to overcome these problems.

In the future, we foresee the need to provide a form of guided exploration, where visual cues help to perform a top-down search for regions containing large intensity changes. This would speed up the work of the radiologist, as it allows him to skip over areas where no changes are detected. Also, from a radiological perspective, there is a high demand to further extend the measurement capabilities of the application. A physical measurement of the volume of change would help in tracking the progression.

This study demonstrates the need for visual analysis methods for whole-body MR. Enabling posture-independent visualization in whole-body MR is a key step toward more effective total body oncological evaluation in clinical practice.

## Footnotes

1 For retrospective anonymized studies from routine patient care in Dutch University Medical Centers, institutional review board approval is not required.
